# The Adaptor Function of TRAPPC2 in Mammalian TRAPPs Explains TRAPPC2-Associated SEDT and TRAPPC9-Associated Congenital Intellectual Disability

**DOI:** 10.1371/journal.pone.0023350

**Published:** 2011-08-15

**Authors:** Min Zong, Xing-gang Wu, Cecilia W. L. Chan, Mei Y. Choi, Hsiao Chang Chan, Julian A. Tanner, Sidney Yu

**Affiliations:** 1 School of Biomedical Sciences, The Chinese University of Hong Kong, Shatin, New Territories, Hong Kong SAR, People's Republic of China; 2 Epithelial Cell Biology Research Center, The Chinese University of Hong Kong, Shatin, New Territories, Hong Kong SAR, People's Republic of China; 3 Department of Biochemistry, University of Hong Kong, Pokfulam, Hong Kong SAR, People's Republic of China; University of South Florida, United States of America

## Abstract

**Background:**

The TRAPP (Transport protein particle) complex is a conserved protein complex functioning at various steps in vesicle transport. Although yeast has three functionally and structurally distinct forms, TRAPPI, II and III, emerging evidence suggests that mammalian TRAPP complex may be different. Mutations in the TRAPP complex subunit 2 (TRAPPC2) cause X-linked spondyloepiphyseal dysplasia tarda, while mutations in the TRAPP complex subunit 9 (TRAPPC9) cause postnatal mental retardation with microcephaly. The structural interplay between these subunits found in mammalian equivalent of TRAPPI and those specific to TRAPPII and TRAPPIII remains largely unknown and we undertook the present study to examine the interaction between these subunits. Here, we reveal that the mammalian equivalent of the TRAPPII complex is structurally distinct from the yeast counterpart thus leading to insight into mechanism of disease.

**Principal Findings:**

We analyzed how TRAPPII- or TRAPPIII- specific subunits interact with the six-subunit core complex of TRAPP by co-immunoprecipitation in mammalian cells. TRAPPC2 binds to TRAPPII-specific subunit TRAPPC9, which in turn binds to TRAPPC10. Unexpectedly, TRAPPC2 can also bind to the putative TRAPPIII-specific subunit, TRAPPC8. Endogenous TRAPPC9-positive TRAPPII complex does not contain TRAPPC8, suggesting that TRAPPC2 binds to either TRAPPC9 or TRAPPC8 during the formation of the mammalian equivalents of TRAPPII or TRAPPIII, respectively. Therefore, TRAPPC2 serves as an adaptor for the formation of these complexes. A disease-causing mutation of TRAPPC2, D47Y, failed to interact with either TRAPPC9 or TRAPPC8, suggesting that aspartate 47 in TRAPPC2 is at or near the site of interaction with TRAPPC9 or TRAPPC8, mediating the formation of TRAPPII and/or TRAPPIII. Furthermore, disease-causing deletional mutants of TRAPPC9 all failed to interact with TRAPPC2 and TRAPPC10.

**Conclusions:**

TRAPPC2 serves as an adaptor for the formation of TRAPPII or TRAPPIII in mammalian cells. The mammalian equivalent of TRAPPII is likely different from the yeast TRAPPII structurally.

## Introduction

Transport protein particle (TRAPP) is a conserved protein complex that functions in secretory and endocytic pathways [1]. This complex was first identified in yeast [2] and it is now clear that distinct forms of the TRAPP complex may exist for specific functions [3], [4], [5], [6]. A total of ten subunits have been identified in yeast, including Trs20, Trs23, Trs31, Trs33, Trs65, Trs85, Trs120, Trs130, Bet3 and Bet5. Additional factors, such as a homolog of Trs20 called Tca17, may also contribute to the formation and functions of TRAPP [7], [8]. Three forms of TRAPP complex have been identified in yeast so far. The subunits Trs20, Trs23, Trs31, Trs33, Bet5 and two copies of Bet3 form a six-subunit core, which contains a total of seven proteins [9]. This six-subunit core is TRAPPI and has intrinsic guanine nucleotide exchange activity for the small GTPase Ypt1p/Rab1 [10]. The two Bet3 subunits within this complex interact specifically with COPII coat component Sec23 [11], thus facilitating the tethering function of TRAPPI. TRAPPII contains all the subunits of TRAPPI with three additional subunits, Trs65, Trs120 and Trs130 [6]. TRAPPII is required for intra-Golgi traffic and endosomal traffic in yeast but the precise mechanism(s) of how it carries out these functions remain(s) unknown [2], [4], [12]. An interaction between the mammalian TRAPPII and the COPI coat component has been documented but the functional significance of this interaction remains to be elucidated [13]. Recent evidence suggests the existence of a third form of TRAPP - TRAPPIII, which consists of the six-subunit core (TRAPPI) plus Trs85 [5]. Deletion of the TRS85 gene in yeast caused defects in autophagy [14], [15], suggesting the involvement of TRAPPIII in autophagy.

All TRAPP subunits except Trs65 are conserved from yeast to mammals [1], [16]. Recently, a distantly related human sequence C5orf44 (FLJ13611) was identified as the human ortholog to Trs65. This sequence was shown to interact with a number of known mammalian TRAPP subunits [17]. In mammals, it appears that TRAPPII is the predominant form but subcellular localization suggests diverse functions [13], [18]. Mammalian TRAPP complex subunit 3 (TRAPPC3), the homologue of Bet3, is localized largely to ER exit sites [19], consistent with its unique function binding to Sec23. Other subunits localize to various structures along the early secretory pathway including TRAPPC10 (Trs130 in yeast) at the cis-Golgi [13], TRAPPC9 (Trs120 in yeast) at the ER exit sites [20], the ER-Golgi intermediate compartments (ERGIC) and cis-Golgi (unpublished data) and TRAPPC4 (Trs23 in yeast) on COPI coated vesicles [13]. COPI vesicles bud from ERGIC and carry cargo proteins to the cis-Golgi [21]. This suggests that mammalian TRAPPII also functions in the early secretory pathway in mammalian cells. In particular, the tethering of COPII vesicles *in vitro* is mediated by mammalian Bet3 [19], and activation of Rab1 by immuno-isolated TRAPPII complex has been demonstrated [13].

TRAPPII differs from TRAPPI by three additional subunits, Trs65, Trs120 and Trs130. Recently cryo-EM images of purified yeast TRAPPII showed that Trs120 and Trs130 bind to opposite sides of the six-subunit core, and evidence of Trs120-Trs130 interaction is not documented in this report [18]. It has been demonstrated that their mammalian homologues TRAPPC9 and TRAPPC10 have interaction [13], although how these two subunits interact with the six-subunit core remains to be determined. It is possible that the EM data provided too low a resolution to show this interaction. Furthermore, the presence of Trs65 is evident in the yeast TRAPPII structure and the main function of Trs65 is to mediate dimerization of the two TRAPPII complexes. It is questionable whether TRAPPII is required to function as a dimer *in vivo* because Trs65 deletion changed the apparent molecular weight of TRAPPII from 1000 kDa to 600 kDa in yeast [17] without causing a block in secretion [6]. In mammals, it has been generally assumed that TRAPPII equivalent only has two additional subunits, TRAPPC9 and TRAPPC10, because the role of putative mammalian Trs65, C5orf44, in TRAPPII assembly has not been well characterized. It is highly questionable that its dimerization function is conserved in C5orf44, because mammalian TRAPP has an apparent molecular weight of 600 kDa in a gel filtration column, identical to the size of yeast TRAPPII isolated in Trs65-deleted strain.

In its primary amino acid sequence, the identifiable domain of Trs120 resides in the carboxyl terminal part of the yeast molecule, but this domain is present in the amino terminus of the mammalian Trs120 (TRAPPC9). This suggests that Trs120 is structurally distinct between yeast and mammals, and such structural distinction will likely manifest in the mammalian TRAPP complex. Further, TRAPPC9 interacts with nuclear factor κB (NFkB)-inducing kinase (NIK) and IkB kinase ß (IKK-ß) [22]. This interaction potentiates the NFkB signaling pathway. As the NFkB signaling pathway does not exist in yeast, this suggests that Trs120 has acquired novel functions during the course of evolution.

The mammalian homolog of Trs85 (TRAPPC8) is also distantly related to the yeast sequence. Yeast Trs85 is thought to be an autophagy-specific subunit for TRAPPIII. Its main function is to target TRAPPIII to autophagosomes, so that TRAPPIII can recruit and activate Ypt1p on autophagosomal membranes. However, the possibility that Trs85-associated autohphagy defect is a secondary consequence of its impaired function in ER to Golgi trafficking has not been ruled out [17]. Nonetheless, TRAPPC8 has been demonstrated to be required for autophagy in mammalian cells. siRNA depletion of Trs85 resulted in reduced autophagy as determined by the extent of lipidation of LC3 [23]. However, the existence of an equivalent of TRAPPIII in mammalian cells has not been established.

Mutations in TRAPP subunits TRAPPC9 and TRAPPC2 have been identified in patients with mental retardation and X-linked spondyloepiphyseal dysplasia tarda (SEDT), respectively [24], [25], [26], [27]. TRAPPC9 mutations result in truncation of the protein. For example, c. 1322 C>T (GenBank Accession #NM_031466), results in nonsense R475X [25], [26]. Another mutation, R570X, was identified independently by Philippe et al [24]. Furthermore, a four-nucleotide deletion results in a stop codon after the codon encoding leucine 772 [26], henceforth known as L772Δ. Some of these truncated TRAPPC9 proteins showed reduced activation of NFκB pathways [24], [26], although impaired vesicle trafficking has not been ruled out. Its homologue TRS120 gene is essential in yeast, and therefore, TRAPPC9 is likely essential. Truncation of this protein could still preserve its major function, but defects in vesicle trafficking may impair neuronal development, leading to its associated intellectual disabilities. Therefore, it is important to characterize how truncation mutants of TRAPPC9 affect the structure and function of TRAPPII in the early secretory pathway.

Mutations in the TRAPPC2 gene locus have been identified in patients with X-linked spondyloephiphyseal dysplasia tarda (SEDT), and therefore TRAPPC2 was also named sedlin [27]. Of the disease-causing mutations identified so far, the majority result in early termination during translation, leading to degradation of partially translated peptides [27], [28], [29], [30], [31], [32], [33], [34]. Of four missense mutations identified in TRAPPC2, recent biochemical analysis revealed that three of these mutations, S73L, F83S and V130D, caused misfolding of the mutant protein, invoking the protein degradation pathway when the GFP-tagged mutant proteins were overexpressed in COS cells [35]. Furthermore, these mutants also failed to bind to some of the known interacting proteins of TRAPPC2, including Myc promoter-binding protein 1 (MBP1), pituitary homeobox 1 (PITX1) and steroidogenic factor 1 (SF1) [28]. As mutant TRAPPC2 proteins are more prone to degradation than the wildtype protein, we suspect that the loss of interaction between mutant TRAPPC2 and its binding proteins is due to protein degradation. The remaining TRAPPC2 mutant, D47Y, is not as extensively degraded as the other three TRAPPC2 mutants. Except a slightly higher affinity to Bet3 in vitro [35], the reason why D47Y causes SEDT has not been thoroughly explored.

In this paper, we identify TRAPPC2 as the major adaptor protein for the interaction between the six-subunit TRAPP core and TRAPPC9 and/or TRAPPC8. Missense mutation, D47Y, showed dramatically reduced binding to TRAPPC9 and TRAPPC8, suggesting the formation of TRAPPII or TRAPPIII is impaired in SEDT patients. Furthermore, we also determined the effect of disease-causing mutations in TRAPPC9 on the formation of TRAPPII complex by studying the ability of TRAPPC9 mutants to bind to TRAPPC2 and TRAPPC10.

## Results

### TRAPPC2 interacts with TRAPPC9

**Figure 1:**
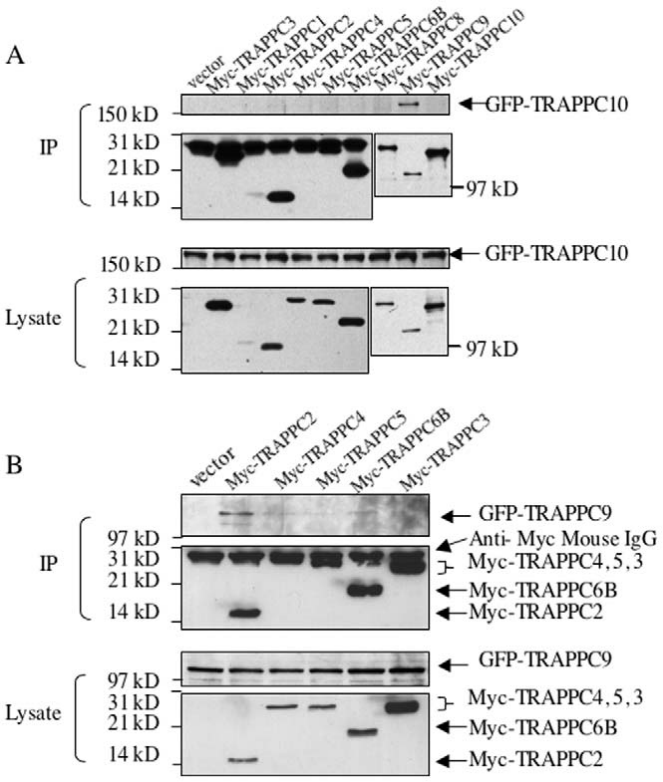
Interactions between putative TRAPPII specific subunits and TRAPPI core subunits. A. GFP-tagged TRAPPC10 was co-transfected with the indicated Myc-tagged TRAPPI core subunits in COS cells for co-IP with antibody against c-Myc. The presence of pulled down TRAPPC10 was detected by immunoblotting with anti-GFP antibody (top panel). The amount of precipitated Myc-tagged proteins is shown in second panel from the top. The levels of protein expression in the transfected lysates are shown in bottom two panels. B. GFP-tagged TRAPPC9 and indicated Myc-tagged TRAPPI core subunits were co-transfected into COS cells. Co-IP and subsequent detection by immunoblotting was performed as above. The experiments shown are representatives of three independent experiments.

We first investigated which subunits of the six-subunit core bind to TRAPPII specific subunits TRAPPC9 and TRAPPC10. TRAPPC10 was previously shown to interact with TRAPPC9 in the mammalian system, but was shown to directly contact the six-subunit core complex in yeast TRAPPII. In mammalian cells, it has not been determined whether other subunits can also bind to TRAPPC10. To test the interactions, we transfected Myc-tagged cDNA encoding various TRAPP subunits together with GFP-TRAPPC10, immunoprecipitated the Myc-tagged proteins with anti-Myc IgG and determined if GFP-TRAPPC10 was present in the pull-downs. As shown in Figure 1A, TRAPPC10 interacts only with TRAPPC9 (top panel, Figure 1A). Although other TRAPP subunits may interact very weakly with TRAPPC10, these interactions were not consistently observed like TRAPPC9. Therefore, we conclude TRAPPC10 interacts only with TRAPPC9. This result suggests that mammalian TRAPPC10 may not bind to the six-subunit core the same way as the yeast complex. As TRAPPC10 only binds to TRAPPC9, we therefore hypothesized that TRAPPC9 binds directly to the six-subunit core, bringing along TRAPPC10 to the complex. To test this, we repeated the co-immunoprecipitation (co-IP) experiment by co-transfecting GFP-TRAPPC9 with various Myc-tagged TRAPP subunits. As shown in Figure 1B, TRAPPC2 and to a lesser extent, TRAPPC6B (Trs33B in yeast), were able to specifically pull down TRAPPC9 (Figure 1B, top panels). Here, we have established that a direct interaction between TRAPPC2 and TRAPPC9 is the physical link between the six-subunit core and the TRAPPII-specific subunits in mammalian TRAPP. TRAPPC10 is indirectly associated with the core via TRAPPC9.

### TRAPPC2 interacts with TRAPPC8

**Figure 2:**
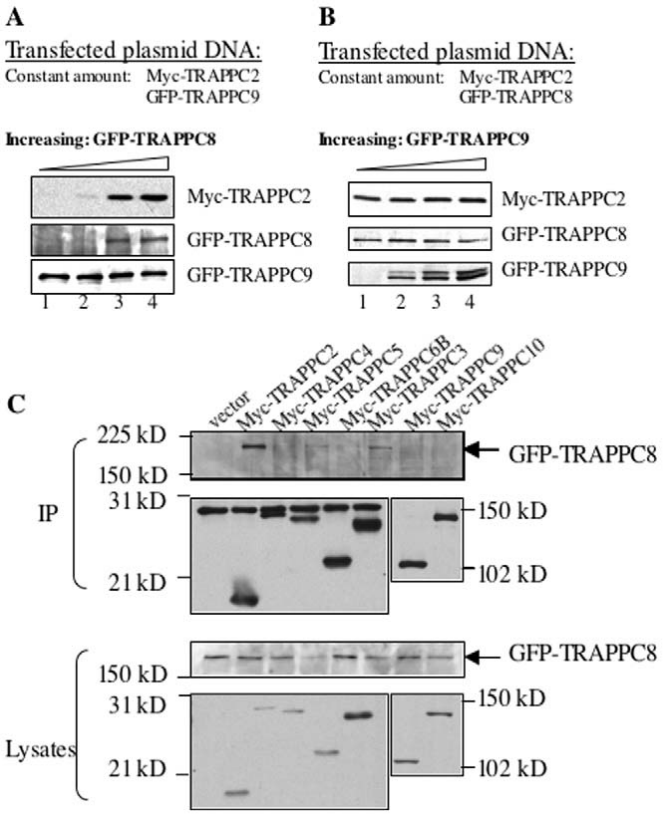
TRAPPC8 interacts mainly with TRAPPC2. A. Increasing amounts of GFP-TRAPPC8 cDNA were co-transfected with a constant amount of Myc-TRAPPC2 and Myc-TRAPPC9. The levels of protein expression of all three TRAPP subunits were determined by immunoblotting the lysates of the transfected cells. B. Increasing amounts of GFP-TRAPPC9 cDNA were co-transfected with a constant amount of Myc-TRAPPC2 and Myc-TRAPPC8. C. GFP-tagged TRAPPC8 and the indicated Myc-tagged TRAPPI core subunits were co-transfected into COS cells. Co-IP and subsequent detection by immunoblotting was performed as in Figure 1. The experiments shown are representatives of three independent experiments.

Although mammalian TRAPPC8 has been postulated to be a subunit of TRAPP in a complex that functions similarly to the yeast TRAPPIII, mammalian TRAPPIII has never been demonstrated to exist. To systematically test how TRAPPC8 binds to TRAPPI to form the mammalian equivalent of TRAPPIII, we decided to test the interaction of TRAPPC8 with individual TRAPP subunits. During the course of establishing conditions for transfecting various DNA constructs to COS1 cells for overexpression, we noticed that TRAPPC2 protein expression was elevated when the same cells were co-expressed with TRAPPC8, but not with TRAPPC9. A careful determination of this effect is demonstrated in Figure 2. When an increasing amount of GFP-TRAPPC8 cDNA was co-transfected with constant amount of Myc-TRAPPC2 cDNA, the level of Myc-TRAPPC2 protein increased (Figure 2A, top panel). This effect was not observed when we tested another TRAPP subunit, TRAPPC9. An increased amount of GFP-TRAPPC9 did not increase the protein expression level of the co-transfected Myc-TRAPPC2 cDNA (Figure 2B, top panel). These results suggest that TRAPPC8 is directly interacts with TRAPPC2. When degradation-prone mutants of Myc-TRAPPC2 were co-transfected with GFP-TRAPPC8, the expression level of these mutants significantly increased as compared to samples co-transfected with GFP-TRAPPC9 (Supplementary Figure S1), suggesting that TRAPPC8 can slow the degradation of TRAPPC2. This finding is not altogether surprising in view of the fact that TRAPPC8 has been demonstrated to be involved in autophagy.

**Figure 3:**
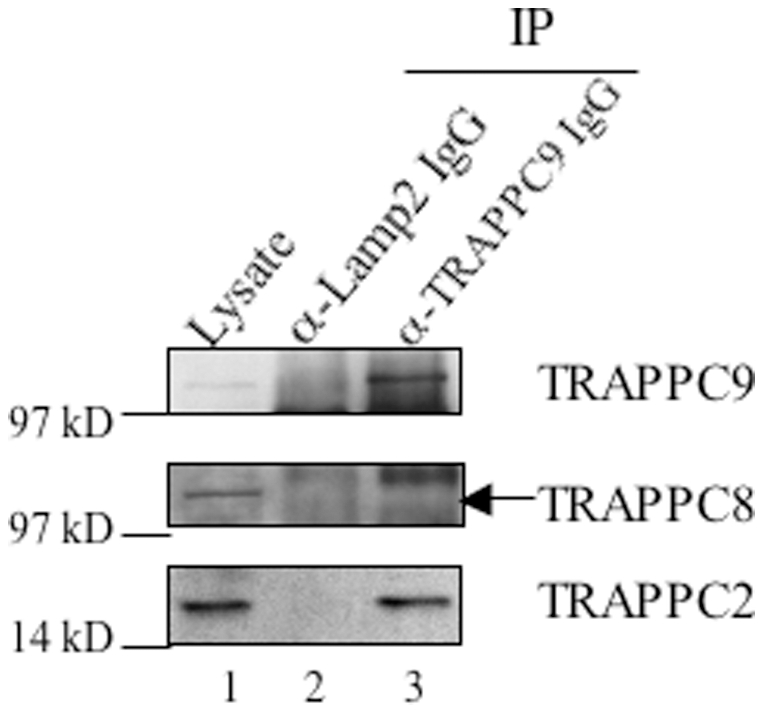
Native TRAPP complex containing TRAPPC9 does not contain TRAPPC8. Native TRAPP complex was isolated by IP using antibody against TRAPPC9 (lane 3). The presence of TRAPPC9 (top panel), TRAPPC2 (bottom panel) and TRAPPC8 (middle panel) was detected by immunoblotting using antibodies specific to these proteins. IP using antibody against lysosomal marker LAMP2 serves as negative control (lane 2). HEK293 lysate used in this experiment (approximately 1% of input) is shown in lane 1. The experiment shown is a representative of two independent experiments.

In cells co-transfected with GFP-tagged TRAPPC8 and various Myc-tagged TRAPP subunits, we performed a co-IP experiment using antibody against c-Myc and determined whether any TRAPP subunit can pull down GFP-TRAPPC8. TRAPPC2 and TRAPPC3 were able to pull down GFP-TRAPPC8, with TRAPPC2 having a stronger interaction (Figure 2C). These results suggest that TRAPPC2 serves as the adaptor that links either TRAPPC9 or TRAPPC8 to the core resulting in the mammalian equivalents of TRAPPII and TRAPPIII, respectively. If this hypothesis is correct, we will expect that TRAPPC2 cannot bind to both TRAPPC8 and TRAPPC9 at the same time. To test this notion, we performed immunoprecipitation using antibody against TRAPPC9 to isolate endogenous TRAPPC9-containing TRAPP complex, and determined whether TRAPPC8 is present in the isolated TRAPP complex (Figure 3). Anti-TRAPPC9 antibody (Figure 3, lane 3), but not control antibody against LAMP2 (Figure 3, lane 2), could immunoprecipitate endogenous TRAPP complex from HEK293 cell lysate, as indicated by the presence of TRAPPC2. TRAPPC8, however, was not present in this TRAPPC9-positive TRAPP complex, confirming TRAPPC9 and TRAPPC8 do not co-exist in the same TRAPP complex in vivo.

### A disease-causing mutant of TRAPPC2 fails to interact with either TRAPPC9 or TRAPPC8

**Figure 4:**
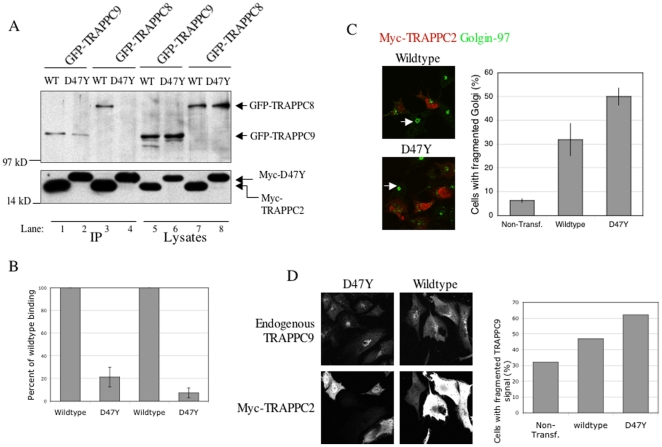
TRAPPC2(D47Y) mutant shows a reduced binding capacity for TRAPPC9 and TRAPPC8. A. Lysates from co-transfections of GFP-TRAPPC9 or -TRAPPC8 with either Myc-TRAPPC2 or -TRAPPC2(D47Y) were subjected to IP using anti-Myc antibody. Wildtype Myc-TRAPPC2 was able to pull down GFP-TRAPPC9 (lane 1) and GFP-TRAPPC8 (lane 3). D47Y mutant, however, showed drastically reduced ability to pull down GFP-TRAPPC9 (lane 2), and failed to pull down GFP-TRAPPC8 (lane 4). The expression levels of these proteins are shown in lanes 5–8. B. A quantification of the degree of binding was performed using data collected from three experiments similar to the one shown in A. The intensity of the TRAPPC8 or TRAPPC9 co-precipitated by D47Y is expressed as a percentage of the wildtype TRAPPC2 binding in each co-IP experiment. Error bars = S.E.M. C. COS cells transfected with wildtype or D47Y Myc-tagged TRAPPC2 were stained with antibodies against Golgin-97 and AF488-secondary antibody (green signal, left panels). Myc-TRAPPC2 expression was detected with TRITC-conjugated 9E10 antibody (red signals, left panels). Golgi fragmentation was quantified by counting cells with fragmented Golgin-97 signals (right panel). In three independent experiments, the total number of cells counted is 352 (non-transfected), 277 (wildtype) and 323 (D47Y). Error bar = S.E.M. D. CHO cells transfected with wildtype or D47Y Myc-tagged TRAPPC2 were stained with antibodies against TRAPPC9 and AF488-secondary antibody (top left panels). The expression of Myc-tagged TRAPPC2 was detected by TRITC-9E10 (red) (bottom left panels). The ability of wildtype or D47Y to disrupt the native localization of TRAPPC9 was quantified using images obtained similarly to those shown in the top panels (right panel). A total of 119, 98 and 149 cells were counted in D47Y, wildtype TRAPPC2, and non-transfected CHO cells, respectively.

We recently characterized four missense mutations of TRAPPC2 [35]. Three of these mutations, S73L, F82S and V130D, caused degradation of the mutant proteins. The other missense mutation, D47Y, did not cause extensive protein degradation but has been implicated to affect the TRAPP complex dynamics because there is a change in the binding affinity of this mutant to TRAPPC3. After identifying TRAPPC2 as the binding partner of TRAPPC9 and TRAPPC8, we wanted to test whether the D47Y mutant impairs the interaction with either of these proteins. When the Myc-tagged wildtype or D47Y TRAPPC2 cDNA was co-expressed with GFP-tagged TRAPPC9 or TRAPPC8, wildtype TRAPPC2 was able to pulldown TRAPPC9 or TRAPPC8 (Figure 4A, lanes 1 and 3, respectively). However, D47Y showed a dramatically reduced affinity for TRAPPC9 and TRAPPC8 (Figure 4A, lanes 2 and 4, respectively). In this experiment, we adjusted the level of protein expression of TRAPPC2 proteins by the amount of DNA used in our transfection. The levels of protein expression for these proteins are comparable in the lysates used for co-immunoprecipitation (Figure 4A, lanes 5–8). When the degree of binding by D47Y and by wildtype TRAPPC2 was quantified, it was clear that D47Y had about 21% and 7% of the wildtype protein for its affinity to interact with TRAPPC9 and TRAPPC8, respectively (Figure 4B). This result indicates that the adaptor function of TRAPPC2 is compromised in the D47Y mutation. D47Y may impair the formation of TRAPP complex(es) that contain(s) TRAPPC9 or TRAPPC8, causing the SEDT phenotype in patients with this mutation. To investigate the effect of D47Y in vivo, we overexpressed this mutant in COS cells and determined its effect on the Golgi (Figure 4C, left panels). Golgi marker Golgin-97 appears as a large dot-like structure in COS cells (arrows, Figure 4C, left panels). However, the signal is fragmented in cells expressing either wildtype or D47Y mutant of TRAPPC2. Upon careful quantification, we observed that D47Y mutant had a more disruptive effect on the integrity of the Golgi than wildtype protein. 50% of the D47Y overexpressing cells have fragmented Golgi, as compared to 32% and 6.2% for wildtype TRAPPC2 overexpressing cells and non-transfected cells, respectively (Figure 4C, right panel). TRAPPC9 localization was also disrupted by D47Y more severely than wildtype protein (Figure 4D). 62% of the cells overexpressed with D47Y having fragmented TRAPPC9 localization, compared to 47% cells with overexpression of wildtype TRAPPC2 (Figure 4D, right panel). These results indicate that D47Y mutant, when overexpressed, can act as a dominant negative fashion to interfere with the assembly of TRAPPII or TRAPPIII.

### Disease-causing mutations in TRAPPC9 reduce the binding affinity of the mutant protein to TRAPPC10 and TRAPPC2

**Figure 5:**
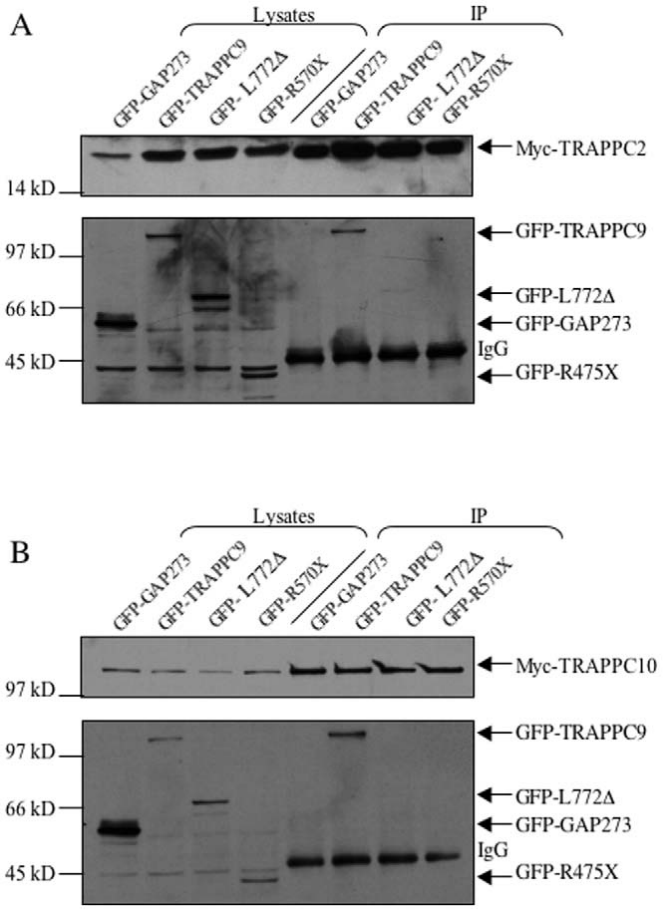
Disease-causing deletional mutants of TRAPPC9 fail to bind to TRAPPC2 or TRAPPC10. A. GFP -GAP273, -TRAPPC9, -TRAPPC9(L772Δ) and -TRAPPC9(R475X) were tested for the interaction with Myc-tagged TRAPPC2. The levels of protein expression in the transfected lysates are shown in lanes 1 to 4. Lanes 5 to 8 are immunoprecipitants. Full-length TRAPPC9 was efficiently pulled down by Myc-TRAPPC2 (lane 6), but not the deletion mutants of TRAPPC9 (lane 7 and 8), or negative control GFP-GAP273 (lane 5). B. Full-length and deletional mutants of TRAPPC9 were tested for the interaction with Myc-tagged TRAPPC10. The levels of protein expression in the transfected lysates are shown in lanes 1 to 4. Lanes 5 to 8 are immunoprecipitants. Full-length TRAPPC9 was efficiently pulled down by Myc-TRAPPC10 (lane 6), but not the deletional mutants (lane 7 and 8), or negative control GFP-GAP273 (lane 5). The experiments shown are representatives of two independent experiments.

Another genetic disease caused by mutations in TRAPP subunits is a subset of intellectual disability with postnatal microcephaly. Mutations in TRAPPC9 have been isolated in susceptible individuals. We characterized the effect of two such mutations in TRAPPC9 with respect to their abilities to bind to TRAPPC2 and TRAPPC10. The mutations tested result in truncation of the protein (see Introduction). In a co-IP experiment similar to the ones described in Figure 1, we detected a drastic reduction in the interaction between mutant TRAPPC9 and TRAPPC2 (Figure 5A). Both GFP-tagged TRAPPC9 mutant proteins have slightly higher but comparable levels of protein expression to the GFP-tagged wildtype protein when expressed in COS1 cells, but the affinity of mutant L772Δ toward TRAPPC2 is drastically reduced as compared to wildtype protein. For R475X, its affinity to TRAPPC2 is reduced to a level below the detection limit of this experiment. Myc-TRAPPC2 failed to pull down negative control protein GFP-GAP273, a GFP fusion protein containing the carboxyl terminus 273 residues of ARFGAP1 [36], [37]. ARFGAP1 does not interact with the TRAPP complex (unpublished data). These mutants also failed to bind to TRAPPC10 as Myc-TRAPPC10 could not pull down either R475X or L772Δ (Figure 5B). These results suggest that TRAPPC9 interacts with TRAPPC2 and TRAPPC10 at the carboxyl terminus of the protein that is downstream of leucine 772.

## Discussion

**Figure 6:**
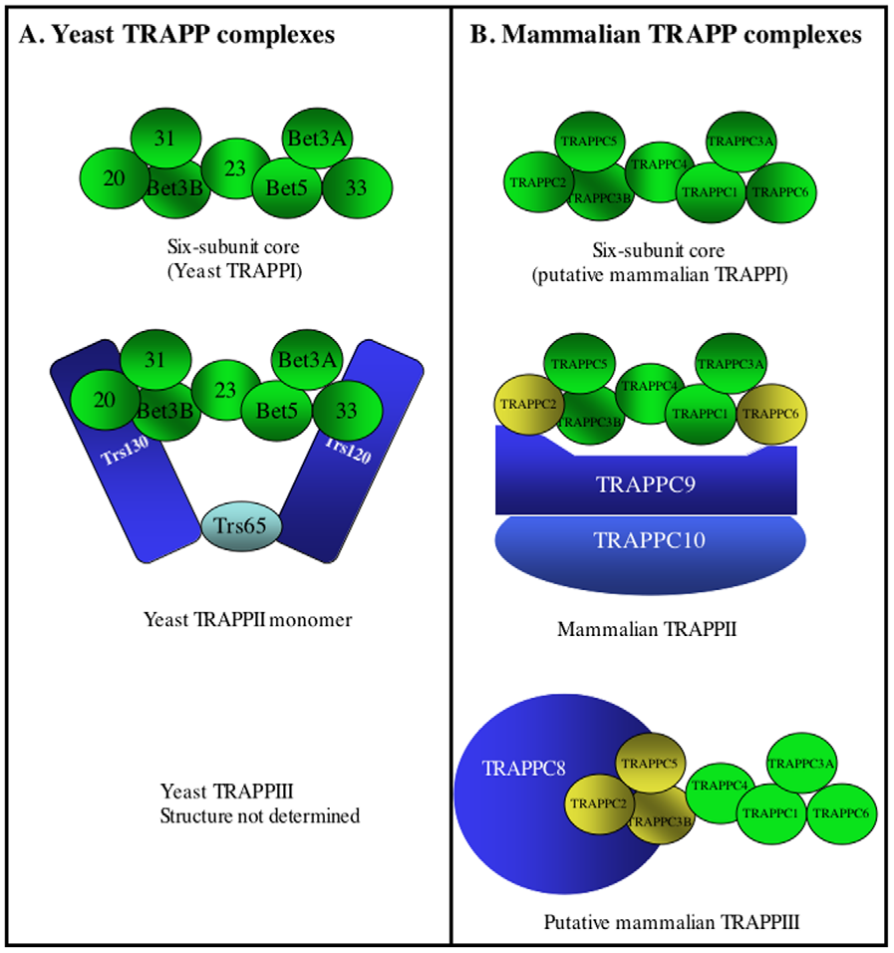
Schematic diagram of the yeast and mammalian TRAPP complexes. A. Schematic diagram of yeast TRAPP complexes. B. Schematic diagram of the putative mammalian TRAPP complexes. The subunits that interact with TRAPPII- or TRAPPIII- specific subunits are labeled in yellow.

The present report describes the role of TRAPPC2 as an adaptor for the TRAPP complex in mammalian cells, mediating interactions with both TRAPPC9 and TRAPPC8. Given the small size of mammalian TRAPPC2 (16 kDa), we expected this subunit could not simultaneously interact with both TRAPPC9 and TRAPPC8. Indeed, TRAPP complex isolated by immunoprecipitation with antibody against TRAPPC9 was devoid of detectable TRAPPC8, suggesting TRAPPC2 cannot bind to both proteins at the same time. This biochemical property seems to be conserved from the yeast protein as mass spectrometry analysis of HA-tagged TRAPPC8 immuno-isolated protein complex did not recover any sequence of TRAPPII-specific subunits [17]. TRAPPC2 is not required for Ypt1p/Rab1 GEF activity, making it the ideal subunit to serve as an adaptor for the association with TRAPPII or TRAPPIII specific subunit. Weak but observable interaction between TRAPPC6B and TRAPPC9 was also detected, suggesting that contacts between TRAPPC9 and the six-subunit core complex are more extensive than just directly with TRAPPC2. Our result is only partially consistent with a recent report suggesting that yeast Trs120 interacts with the six-subunit core complex via Bet3-Trs33 side of the core, whereas Trs130 interacts with the six-subunit core from the opposite side where Trs20 is located [18]. This report analyzed the organization of yeast TRAPPII complex by single particle EM. We hypothesize that TRAPPC9 somehow wraps around the six-subunit core with contact points at residues from TRAPPC2 and TRAPPC6B, and possibly other subunits whose interactions with TRAPPC9 are too weak to be detected in our assay system. Our interaction studies, therefore, indicate that the possible locations of TRAPPC9 and TRAPPC10 relative to the six-subunit core are slightly different from the yeast TRAPPII structure (Figure 6). There has likely been an evolutionary divergence between yeast Trs120 and Tra130 and their human counterparts resulting in a slightly different TRAPPII structural organization.

Such notion is supported by the fact that the conserved domain in mammalian TRAPPC9 is at the carboxyl-terminus, whereas the same domain in yeast Trs120 is at the amino-terminus. This Trs120 conserved domain (pfam: 08626; CDD:149620) contains approximately 200 to 300 residues depending on the species. In the human sequence (gi:190359999), the conserved domain encompasses residues 606 to 923, whereas in yeast *S. cerevisiae*, the corresponding domain is found in the amino-terminal 186 residues of the protein (gi:74610391). No homology is found outside this domain.

Supporting our hypothesis that TRAPPC2 is a subunit serving an adaptor-like function for linking TRAPPC9 or TRAPPC8 to the six-subunit core is the observation that a disease-causing mutation of TRAPPC2, D47Y, is incapable of interacting with both TRAPPC9 and TRAPPC8. This result suggests that D47 is at or very near the point of interaction. Structurally, D47 is a conserved residue that is exposed on the surface of the protein implicated in protein-protein interactions [9], [35]. It has been previously demonstrated that a loss of TRAPPC2 function, due to misfolding and degradation of the mutant TRAPPC2 protein, cause SEDT. In the present study, we have provided evidence the impairment of TRAPPII and/or TRAPPIII formation and their associated functions could be the cause of SEDT. In a similar experiment, we have further identified that the carboxyl terminus of TRAPPC9 is required for its interaction with TRAPPC2 and TRAPPC10, as deletional mutants of this domain found in some patients with intellectual disability failed to interact with TRAPPC2 or TRAPPC10. This suggests that in patients suffering from TRAPPC9-associated congenital intellectual disability, TRAPPII function must be compromised.

Taken together, mammalian TRAPPC2 serves as adaptor for the formation of the mammalian equivalents of TRAPPII and TRAPPIII by interacting with TRAPPC9 and TRAPPC8, respectively. This finding provides a biochemical explanation to the disease causes of SEDT and TRAPPC9-associated congenital intellectual disability.

## Materials and Methods

### DNA

We ordered PCR primers specific for the cDNAs of various mammalian TRAPP subunits and performed PCR amplification of these sequences for subcloning. The sequences were subcloned into pCMV-Myc (Clontech) or peGFP-Cx vector (Clontech) for mammalian expression. The reading frames of these genes were maintained to ensure Myc-tagged or GFP-tagged fusion proteins will be expressed after transfection. Sequencings of the resulting constructs were performed to confirm no PCR-generated mutation in the sequences. Of note, we used an isoform of human TRAPPC9 that contain 944 amino acid residues. The sequence corresponds to the carboxyl terminal 944 residues of TRAPPC9 (NM_001160372.1) and uses Met249 as start codon. This sequence was originally identified along with the cloning of a nearby gene KCNK9 potassium channel by exon trapping [38].

### Antibodies

Mouse monoclonal antibody against c-Myc, 9E10 (sc-40), 9E10-conjugated with TRITC, and rabbit polyclonal antibody against GFP (sc-8334) were purchased from Santa Cruz Biotechnology, Inc. (CA, USA). Monoclonal antibody against Golgin-97, CDF4, was a gift from Dr. Wing Keung Liu's laboratory (The Chinese University of Hong Kong), and was originally purchased from Invitrogen. Rabbit polyclonal antibody against TRAPPC9, TRAPPC2 and TRAPPC8 were developed by immunizing rabbits with bacterially overexpressed and purified recombinant proteins. For TRAPPC2, His_6_-tagged full-length protein was used for immunization. For TRAPPC8, a fragment from residue 1286 to 1439 (totally 153 residues) fused with the maltose binding protein (MBP) was purified and used for immunization. For TRAPPC9, a fragment of 300 residues from the carboxyl terminus of the protein was fused with GST and the fusion protein was used for immunization. Antisera generated from these immunizations were collected after three or four immunity boosts. Antibodies specific to TRAPPC9 were purified by incubating the 2–3 ml of anti-serum with a piece of nitrocellulose membrane containing 1 mg of antigen protein for one hour at room temperature. After brief washes, the bound antibodies were eluted from the nitrocellulose membrane by 0.1 M glycine, p.H. 2.0. The eluted antibody solution was neutralized with appropriate amount of Tris, p.H. 8.8, dialyzed against PBS and concentrated for storage.

### Cells and culture media

COS1 cells, originally from the ATCC, were maintained in Debucco's minimum essential medium (DMEM) (Invitrogen) supplemented with 10% fetal bovine serum (FBS) (Invitrogen).

### Sequence analysis

Sequence analysis was performed using the Homologene database from NCBI.

### Transfection

Transfections of COS1 cells were carried out using polyethylenimine (PEI) method [39]. Briefly, for each 10 cm tissue culture plate of COS1 cells, a total of 10 µg of the plasmid DNA was mixed with 30 µg of PEI (branched, 25 kDa, Sigma, 408727) in 0.8 ml of PBS. After 20 minutes, the PEI∶DNA complex was added to the cells and incubated for 5–6 hours. The transfection medium was replaced with fresh medium for an addition 24–36 hours and the transfected cells were harvested for analysis.

### Immunoprecipitation

COS1 cells transfected with the indicated DNA plasmids were lysed with 1 ml of lysis buffer [20 mM Tris, pH 8.0, 100 mM NaCl, 0.1% NP-40 and 1× protease inhibitor cocktail Complete® (Roche)]. The cells were scrapped from the 10 cm plates and collected in 1.5 ml tubes. After brief vortex, the lysates were centrifuged at 7600 g at 4°C for 10 minutes. The supernatants were collected and subjected to immunoprecipitation using 1–2 µg of anti-c-Myc antibody and 20 µl of protein-A sepharose slurry (Sigma, P3391). After at least 3 hours of incubation at 4°C with agitation, the immunoprecipitants were washed three times with lysis buffer and once with 20 mM Tris, pH 8.0 and 100 mM NaCl. 30 µl of 2× SDS sample buffer were added to each immunoprecipitant and the samples were subjected to SDS-PAGE and immunoblotting analysis. For immunoprecipitation of TRAPP complex with anti-TRAPPC9 antibody, lysates generated from approximately 2×10^7^ HEK293 cells were subjected to immunoprecipitation using antibody against TRAPPC9, or against LAMP2 (as control). The rest of the procedures are identical to description mentioned above. For quantification of immunoblot data, the X-ray film was first scanned in a Bio-Rad GS-800 densitometer. The intensity of the signals was quantified by AlphaEaseFC 4.0 (Alpha Innotech Corp.)

### Fluorescence microscopy

#### Immunofluorescence staining

COS or CHO-K1 cells, seeded in 12 mm glass cover slips and transiently transfected with Myc-tagged TRAPPC2, were fixed with −20°C methanol for 5 minutes, re-hydrated and blocked with PBS plus 1% BSA for 20 minutes. Then the cells were stained with the indicated antibodies at concentration of 1 µg/ml diluted in PBS plus 1% BSA. After 30 minutes incubation, the cells were washed three times with PBS plus 1% BSA. Goat anti- mouse IgG conjugated with Alexa fluor 568 (1∶1000 dilution) was applied to the samples. After 30-minute incubation, the samples were washed 4 times before they were mounted on glass slide in Fluoromount-G (Southern BioTech, USA, 0100-01) for microscope visualization.

#### Fluorescence microscopy visualization and image capture

Fluorescence signals were visualized and acquired in a Carl Zeiss LSM-5 laser scanning confocal microscope with 63× objective lens. The images were processed with Adobe Photoshop 7.0.

## Supporting Information

Figure S1 **TRAPPC8 protects TRAPPC2 mutant from protein degradation.** TRAPPC9 or TRAPPC8 was co-transfected with various mutants of TRAPPC2 using the same amount of TRAPPC2 cDNA. The relative protein expression levels of the wildtype TRAPPC2 or the indicated mutants were determined by immunoblotting.
(DOC)
