# Supplementary material for: The Adaptor Function of TRAPPC2 in Mammalian TRAPPs Explains TRAPPC2-Associated SEDT and TRAPPC9-Associated Congenital Intellectual Disability
Source: PLoS One. 2011 Aug 15;6(8):e23350. doi: 10.1371/journal.pone.0023350 (PMC3156116; doi:10.1371/journal.pone.0023350)
Supplement: Figure S1 — TRAPPC8 protects TRAPPC2 mutant from protein degradation. TRAPPC9 or TRAPPC8 was co-transfected with various mutants of TRAPPC2 using the same amount of TRAPPC2 cDNA. The relative protein expression levels of the wildtype TRAPPC2 or the indicated mutants were determined by immunoblotting. (DOC) [file pone.0023350.s001.doc]

###### Zong et al 2011

###### Supplementary Figure S1. TRAPPC8 protects TRAPPC2 mutant from protein degradation.

TRAPPC9 or TRAPPC8 was co-transfected with various mutants of TRAPPC2 using the same amount of TRAPPC2 cDNA. The relative protein expression levels of the wildtype TRAPPC2 or the indicated mutants were determined by immunoblotting.
